# 

**Published:** 1829-01-01

**Authors:** 


### MEDICO-CHIRURGICAL

### REVIEW,

AND

### JOURNAL

OF

### PRACTICAL MEDICINE.

*(NEW SERIES.)*

VOLUME TEN,

[1st of OCTOBER, 1828, to 31st of MARCH,]

1829.

VOL. XIV. of ANALYTICAL SERIES.

EDITED BY JAMES JOHNSON, M.D

&c. &c. &c.

103676
727
Surgeon Gen'l's Office
LIBRARY
57443
Washington

LONDON;
PUBLISHED BY S. HIGHLEY, 174, FLEET STREET,
AND WEBB STREET, ST. THOMAS'S HOSPITAL,
And all other Booksellers.

W1
ME 75 T

### BIBLIOGRAPHICAL RECORD.

1. Dissertatio Medica Inauguralis, de Febri Endemicâ Bataviæ, &c. Auctore JOHANNE THOMPSON, Anglo. In Classe Regia Chirurgo, &c. &c. Edinburgi, MDCCCXXVIII.

☞ *An ably written, well-supported thesis.*

---

2. Medical Essays on Fever, Inflammation, Rheumatism, Diseases of the Heart, &c. By JOSEPH BROWN, M.D. of the Royal College of Physicians, one of the Physicians to the Sunderland and Bishopwearmouth Infirmary, &c. 8vo, pp. 309, London. Longman & Co. 1828.

☞ *See the present number.*

---

3. Remarks on the Treatment of the Insane, and the Management of Lunatic Asylums, &c. By E. P. CHARLESWORTH, M.D. one of the Physicians of the Lincoln Lunatic Asylum. 8vo, pp. 40. Sept. 1828.

---

4. A Letter addressed to his Excellency the Earl of Chatham, K.G. Governor of Gibraltar, &c. &c. relative to the Febrile Distempers of that Garrison. By W. W. FRASER, Esq. Inspector of Hospitals, &c.

---

5. An Essay on a New Mode of Treatment for Diseased Joints, and the Non-union of Fracture; with Cases and Formulæ of the various Preparations used. By THOMAS BUCHANAN, C.M. &c. &c. 8vo, pp. 100. Longman's London, 1828.

---

6. Part the First of a Theory concerning the Existence of an Universal Etherial Essence; or, Spirit of Motion, supposed to fill all Space. By a Novice. 12mo, stitched, pp. 46. Dublin, 1828.

Part the Second of Ditto, pp. 50.

---

7. A general Description of the Bones of the Skeleton, intended for the Use of Students. By HENRY KEMP RANDELL, Member of the Royal College of Surgeons. 12mo, pp. 144. Highley, London, 1828.

☞ *This is well executed.*

8. A Stethoscopic Chart, in which may be seen at one View, the Application of Auscultation and Percussion to the Diagnosis of Thoracic Diseases, comprising their chief Pathognomonic Symptoms and the Use of the Stethoscope in other Diseases. Arranged by S. E. HOSKINS, M.R.C.S.

☞ *This is an ingenious contrivance.*

---

9. On Public Health in India. By JAMES RANKIN, M.D. 8vo, pp. 53.

---

10. A Manual on Midwifery; or, a Summary of the Science and Art of Obstetric Medicine; including the Anatomy, Physiology, Pathology, and Therapeutics peculiar to Females; Treatment of Parturition, Puerperal and Infantile Diseases; and an Exposition of Obstetrico-Legal Medicine. By MICHAEL RYAN, M.D. &c. &c. 12mo, pp. 353. London, 1828.

*Lately were received from the same Author.*

11. An Essay on the Supply of Water to the Metropolis, on the Natural, Chemical, and Medical History of Water, being a Guide to all the known Mineral Waters—1828: and a Lecture on the Rise and Progress of Midwifery. London, 1828.

---

12. A Treatise on the Nature and Cure of Intestinal Worms of the Human Body; arranged according to the Classification of Rudolphi and Bremser, and containing the most approved Methods of Treatment, as practised in this Country and on the Continent. By W. RHIND, Surgeon, M.R. M.S.E. Illustrated by Six Plates. 8vo, pp. 152. Highley, London, 1828.

---

13. Traité de Chimie. Par M. DUMAS, Répétiteur à l'Ecole Polytechnique, &c. &c. Tome premier, 8vo, pp. 689. Paris, 1828.

---

14. Traité de Chimie appliquée aux Arts. Par M. DUMAS. Atlas. Tome. 16 planches. Paris, 1828.

15. The Anatomy and Physiology of the Nervous System. By Valentine Flood, A.M. M.B. Member of the Royal College of Surgeons in Ireland, &c. In Two Volumes. Vol. 1, 12mo, pp. 315. Dublin, 1828.

---

16. Elements of General and Pathological Anatomy, adapted to the present State of Knowledge in that Science. By David Craigie, M.D. 8vo, pp. 816. Edinburgh, 1828.

---

17. No. 14 of Flora Medica, containing Botanical Descriptions, Natural History, Chemical Properties and Analyses, Medical Properties, and Uses, &c. &c. of the Officinal Plants, comprised in the London, Edinburgh, and Dublin Pharmacopœiæ. Edited by a Member of the London College of Physicians, F.L.S. and assisted by several Members of a Botanical Society. Price 2s. 6d. Dec. 1st. 1828. London, Callow and Wilson's.

☞ *This number completes the first volume of the Flora Medica, and it is but justice to say that the work has improved, and deserves to be encouraged.*

---

18. An Essay on the Mechanism of Parturition, from the German of C. F. Naegelè, Professor of Midwifery at Heidelberg. By Edward Rigby, M.D. 12mo, pp. 166. London, 1829.

---

19. A Chart of the Diseases of the Skin.

☞ This Chart is in accordance with the arrangement of Rayer, which is chiefly founded on the classification, nomenclature, and distinctive characters of Willan. The definitions are added, and the effects of the fumigating baths on several of these diseases, as observed by Mr. Green during six years practice with this remedy, are also shewn in the last column.

---

20. Transactions of the Medical and Physical Society of Calcutta. Volume the Third. 8vo, pp. 451. Calcutta, 1828.

---

21. The Medical Reporter, or Analysis of the Sciences of Anatomy, Medicine, Surgery, Chemistry, Materia Medica, Pharmacy, Clinical and Obstetric Medicine, &c. &c. No. 1. Price Fourpence. (Weekly.) Anderson, London, 1828.

22. An Inquiry concerning the relative Connexion which subsists between the Mind and the Brain: with Remarks on Phrenology and Materialism, &c. &c. By William Wildsmith, M.R.C.S.L. &c. &c. 8vo, stitched, pp. 74. London, 1828.

---

23. Personal Narrative of a Journey over land from the Bank to Barnes. By an Inside Passenger. To which is added, a Model for a Magazine, being the Product of the Author's Sojourn at the Village of Barnes, during five rainy Days. 12mo, pp. 166. London, 1828.

---

24. Letters on the Study and Practice of Medicine and Surgery, and on the Topics connected with the Medical Profession; addressed to Students and young Practitioners of Medicine, to Parents and Guardians, and the Public in general. By James Wallace, &c. &c. Glasgow, London, and Edinburgh, 1828. 8vo, pp. 210. Underwoods, London.

---

25. Observations on the Nature and Treatment of Cholera: and on the Pathology of Mucous Membranes. By A. Christie, M.D. Madras Medical Establishment; and lately in Medical Charge of the Civil Department in the Southern Mahratta Country. 8vo. pp. 137. Edinburgh, 1828.

---

26. Observations on the History, Use, and Construction of Obturateurs, or Artificial Palates; illustrated by Cases of Recent Improvements. To which are added numerous Cases of Deficiency of the Lower Jaw, Lips, Nose, &c. &c. with the most efficient means of Restoring the Parts Artificially. By James Snell, Surgeon-Dentist, M.R.C.S., &c. Second edition, 8vo. pp. 106, London, 1828.

---

27. A Treatise on Diseases of the Bones. By Benjamin Bell, Fellow of the Royal College of Surgeons, &c. 8vo. pp. 294, with plates, price 7s. 1828.

---

28. Observations on the Nature and Treatment of Fractures of the Upper Third of the Thigh-Bone, and of Fractures of long standing, &c. &c. &c. By Joseph Amesbury, Surgeon to the South London Dispensary, &c. 8vo. pp. 315, with plates, 1828.

29. A System of Human Anatomy; translated from the fourth edition of the French of H. Cloquet, M.D. Professor of Physiology, &c. with notes and corrected nomenclature. By ROBERT KNOX, M.D. F.R.S.E. Lecturer on Anatomy, Fellow of the Royal College of Surgeons &c. Edinburgh. 8vo. pp. 838. Maclachlan and Stewart, Edinburgh, 1828.

---

30. A Pocket Compendium of Anatomy; containing a correct and accurate Description of the Human Body. By E. WILL. TUSON, Lecturer on Anatomy and Physiology, &c. &c. 12mo. in the shape of a pocket-book, pp. 289, London, 1828.

*** *The plan of the present Compendium is new—and its execution good. To the Student attending the Hospitals and Lecture-Rooms, the work will be useful, being readily carried in the hand or the pocket, and looking exactly like an ordinary pocket-book.*

---

31. A Treatise on Nervous Disorders; including Observations on Dietetic and Medicinal Remedies. By THOMAS RICHARDS, Surgeon, 12mo. pp. 154. London, 1828.

*** *We fear that this Treatise is too professional for the populace, and too popular for the profession. It is free from all quackery, and contains no ad captandum appeals to the patient.*

---

32. Chemical Re-Agents, or Tests; and their Application in Analysing Waters, Earths, Soils, Metalliferous Ores, Metallic Alloys, &c. Originally by F. Accum; improved and brought down to the present state of Chemical Science. By WILLIAM MAUGHAM, Surgeon, Lecturer on Chemistry and Materia Medica, &c. 12mo. pp. 452, one plate, London, 1828.

---

34. Pathological and Practical Researches on Diseases of the Stomach, the Intestinal Canal, the Liver, and other Viscera of the Abdomen. By JOHN ABERCROMBIE, M.D. Fellow of the Royal College of Physicians of Edinburgh, &c. 8vo. pp. 396, Edinburgh, 1828.

---

35. A Practical Treatise on Parturition, comprising the attendant circumstances and Diseases of the Puerperal and Pregnant States. By SAMUEL ASHWELL, M.R.C.S., &c.; to which are appended two Papers, the one containing some remarks on Abdominal Surgery, the other on Transfusion; presented by Dr. Blundell, of Guy's Hospital. 8vo. pp. 546, thirteen plates. London, 1827.

---

36. A Supplement to Myology; illustrated by coloured Plates, on a peculiar construction, containing the Arteries, Veins, Nerves, and Lymphatics; the Abdominal and Thoracic Viscera, the Brain, the Ear, and Eye, &c. By E. W. TUSON, Lecturer on Anatomy and Physiology, Member of the Royal College of Surgeons, &c. Nine plates, price 4l. 10s. Callow & Wilson, London, 1828.

*** *We have already, on several occasions, had reason to speak favourably of Mr. Tuson. On the present we cannot resist the pleasure of expressing the very high opinion we entertain of his ability, ingenuity, and industry. These plates do him credit; they are happily conceived and as happily executed. The two representing the brain and gravid uterus in particular, merit the most commendation. To the student we recommend the work as serving, all that such delineations can—the assisting, not the superseding of dissection.*

---

37. Nouvelle Toxicologie, ou Traité des Poisons, et de l'Empoisonnement, sous le Rapport de la Chimie, de la Physiologie, de la Pathologie, et de la Thérapeutique. Par GUÉRINE DE MANVERS, Docteur en Médecine, &c. &c. 8vo. pp. 412, Paris, 1828.

*** *This is a very able work, and we hope to give an analysis of it shortly.*

---

To Dr. BURROWS we have to apologize for not being able to notice his "Reclamation." We were led into an error, it appears, by a passage which we quoted from his work, and which stated that "all the causes, physical and moral, of insanity, have existed since the origin of man." This passage seemed to us to deny the springing up of new causes, from time to time, with the progress of civilisation. But such is not Dr. Burrows' meaning; for, in another place, which escaped our notice, he traces the great source of mental alienation to the progress of civilisation. The mistake or rather misapprehension on our part is not, we imagine, of so much importance as Dr. B. thinks it. It is now, however rectified.

